# Early Detection of Checkpoint Inhibitor-Associated Myocarditis Using ^68^Ga-FAPI PET/CT

**DOI:** 10.3389/fcvm.2021.614997

**Published:** 2021-02-25

**Authors:** Daniel Finke, Markus B. Heckmann, Esther Herpel, Hugo A. Katus, Uwe Haberkorn, Florian Leuschner, Lorenz H. Lehmann

**Affiliations:** ^1^Department of Cardiology, University Hospital Heidelberg, Heidelberg, Germany; ^2^DZHK (German Centre for Cardiovascular Research), Partner Site Heidelberg/Mannheim, Heidelberg, Germany; ^3^Department of Pathology, University Hospital Heidelberg, Heidelberg, Germany; ^4^Department of Nuclear Medicine, University Hospital Heidelberg, Heidelberg, Germany; ^5^Clinical Cooperation Unit Nuclear Medicine, German Cancer Research Center (DKFZ), Heidelberg, Germany; ^6^Translational Lung Research Center Heidelberg (TLRC), German Center for Lung Research (DZL), Heidelberg, Germany; ^7^German Cancer Research Center (DKFZ), Heidelberg, Germany

**Keywords:** checkpoint- inhibitors, cardio-oncology, myocarditis, positron emission tomography, cardiotoxcity

## Abstract

**Objective:** Checkpoint inhibitors (ICIs) have gained importance in recent years regarding the treatment of a variety of oncologic diseases. The possibilities of diagnosing cardiac adverse autoimmune effects of ICIs are still limited. We aimed to implement FAPI PET/CT imaging in detecting ICI-associated myocarditis.

**Methods:** In a retrospective study, FAPI PET/CT scans of 26 patients who received ICIs from 01/2017 to 10/2019 were analyzed. We compared tracer enrichment in the heart of patients without any signs of a cardiac disease (*n* = 23) to three patients with suspected ICI-associated myocarditis. To exclude any significant coronary heart disease, cardiac catherization was performed. All three patients' myocardial biopsies were examined for inflammatory cells.

**Results:** Three patients showed clinical manifestations of an ICI syndrome including myocarditis with elevated levels of hsTnT (175 pg/ml, 1,771 pg/ml, 157 pg/ml). Further cardiological assessments revealed ECG abnormalities, lymphocyte infiltration of the myocardium in the biopsies or wall motion abnormalities in echocardiography. These patients' FAPI PET/CTs showed cardiac enrichment of the marker which was less distinct or absent in patients receiving ICIs without any signs of immunological adverse effects or cardiac impairment (*n* = 23) [Median SUV myocarditis patients: 1.79 (IQR: 1.65, 1.85), median SUV non-myocarditis patients: 1.15 (IQR: 0.955, 1.52)].

**Conclusions:** Apart from the successful implementation of ICIs in oncological treatments, ICI-associated myocarditis is still a challenging adverse effect. FAPI PET/CT may be used in order to identify affected patients at an early stage. Moreover, when integrated into cancer stage diagnostics, it contributes to cardiac risk stratification besides biomarker, ECG and echocardiography.

## Key Points

- **Question:** Is FAPI PET/CT able to diagnose ICI-associated myocarditis?
- **Pertinent findings:** In a cohort study, FAPI PET/CT was applied in 26 cancer patients receiving ICIs. Three patients with evidence of ICI-myocarditis showed elevated SUVs in the myocardium above the median compared to 23 patients without any evidence of myocarditis.
- **Implications for patient care:** FAPI PET/CT might fill the diagnostic gap to diagnose ICI-associated myocarditis.

## Introduction

Based on their groundbreaking effects on cancer, immune checkpoint inhibitors (ICIs) are currently investigated in more than 2,500 clinical studies for almost all types of cancer. Despite the antitumor effects, adverse immune related responses can lead to serious adverse events (1). Among the variety of organ manifestations, ICI-associated myocarditis has shown a high fatality rate of up to 50% (2). Due to the fact that the reported incidence is very low (around 1.4%) and that the phenotype of this novel syndrome is highly variable, a definitive diagnosis is still challenging. Cardiac MRI, ECG and cardiac biomarkers often show an inconsistent pattern. Therefore, myocardial biopsy with the detection of CD3/CD8+ cells is currently considered as the gold standard (3, 4).

Fibroblast activation protein (FAP) is a protease with endopeptidase activity, cleaving at specific postproline bonds (5). It is involved in various biological processes [e.g., wound healing (6), tissue remodeling (7), or tumor growth (8)]. In cardiomyopathies, in particular, FAP belongs to the most upregulated proteins (9).

The discovery of a fibroblast activation protein inhibitor (FAPI) (10) allowed the development of an imaging technique with the use of a radiotracer to image FAP density (11). ^68^Ga-FAPI PET/CT is currently in clinical use to detect malignancies (11–13). In rats, FAPI PET/CT revealed enrichment in the myocardium after experimental induction of myocardial infarction (MI) (14).

To date, a sensitive, non-invasive method for the detection of ICI-associated myocarditis is still missing. The study was designed to determine whether ^68^Ga-FAPI PET/CT imaging can be used in order to detect ICI-associated myocarditis.

## Materials and Methods

### Patients

From 2017 to 2019, 26 patients were treated with PD-1, PD-L1, or CTLA-4 inhibitors at University Hospital Heidelberg because of their malignant disease and received ^68^Ga-FAPI PET/CT scans to asses their cancer stage.

Patients who are treated with ICIs are observed within a close surveillance protocol at the oncology departments. Creatine kinase is assessed on a regular basis. If there are increased levels or clinical signs and symptoms for acute coronary syndrome or heart failure, cardiac biomarkers are evaluated. According to these observations, a detailed cardiological assessment, including catherization with myocardial biopsy, echocardiography and cardiac MRI, is initiated. Clinically, there have been no signs of heart failure or acute coronary syndrome in the non-myocarditis patients as well as no relevant elevations of creatine kinase.

Further, cardiac assessments including cardiac MRI and cardiac catherization were performed in the myocarditis patients. These patients received ^68^Ga-FAPI PET/CT after the suspect of ICI-associated myocarditis was raised. The pathological results have not been accessible at that timepoint.

The study protocol was approved by the ethics committee of the Medical Faculty of University Heidelberg (S-286/2017, S016/2018).

### ^68^Ga-FAPI PET/CT

FAPI PET/CT scans were performed using 122–336 mBq of Gallium 68 (^68^Ga)-labeled fibroblast activation protein inhibitor (FAPI) which was administered intravenously 60 min before examination. The PET/CT scans were performed with a Biograph mCT Flow™ PET/CT-Scanner (Siemens Medical Solutions) using the following parameters: slice thickness of 5 mm, increment of 3–4 mm, soft-tissue reconstruction kernel, care dose. Immediately after CT scanning, a whole-body PET was acquired in 3D (matrix 200 × 200) in FlowMotion™ with 0.7 cm/min. The emission data was corrected for random, scatter and decay. Reconstruction was conducted with an ordered subset expectation maximization (OSEM) algorithm with two iterations/21 subsets and Gauss-filtered to a transaxial resolution of 5 mm at full-width half-maximum (FWHM). Attenuation correction was performed using the low-dose non-enhanced CT data. The quantitative assessment of standardized uptake values (SUV) was done using a region of interest technique.

### Cardiac MRI

Standard CMR was performed supine in a 1.5-T Ingenia (1.5-T) or 3-T Ingenia CX (3-T) whole body scanner (Philips Healthcare, Best, The Netherlands), with a commercial cardiac phased array receiver coil. Following localizing scans, cine long axis 2-, 3-, and 4-chamber views as well as short axis (SAX) cine images covering the whole LV from the anulus of the atrioventricular valves to the apex (8 mm slice thickness, no gap between each slice) were obtained using a breath-hold, segmented-k-space balanced steady-state free precession sequence (bSSFP) employing retrospective ECG or pulse oximetric gating with 35 phases per cardiac cycles for cardiac morphology.

### Data Accession and Analysis

Patient specific data, including ECG and biomarker, was extracted from the electronic medical reports. For ^68^Ga-FAPI PET/CT analysis, 17 segments of the left ventricle were measured based on the anatomic structure regardless of focal signal enrichment. Graphs were built in R version 3.4.4 with inhouse scripting using the shape and RColorBrewer packages. SUVs have been specified as median values and interquartile range.

For histological sections, myocardial biopsies were stained with Hematoxlin and eosin, anti-CD3 and anti-CD8.

## Results

### Clinical Cases of ICI-Associated Myocarditis

Three patients receiving immune checkpoint inhibitors were admitted with suspected autoimmune myocarditis (Table 1).

- A 62-year-old male patient received two doses of Pembrolizumab (200 mg, q3w) to treat melanoma until he was admitted to our cardio-oncology unit due to elevations of hs-troponinT (hsTnT) (39 pg/ml) and NT-proBNP (2,900 ng/l). Symptoms of heart failure or acute coronary syndrome were absent.
- A 70-year-old male patient was treated with Durvalumab (1,500 mg, q4w), a PD-L1 inhibitor monotherapy due to a hepatocellular carcinoma. After two doses, he complained of pronounced shortness of breath, classified as NYHA III-IV. The worsening of the respiratory deficiency required long-term mechanical ventilation, most likely due to an emerging myasthenia-like syndrome. Aside from the myocarditis therapy, the patient was successfully treated with pyridostigmine to improve the weaning of the ventilation.
- A 74-year-old female patient, diagnosed with metastatic adenocarcinoma of the uterus was treated with Pembrolizumab (200 mg, q3w), a PD-1 inhibitor. 9 months after the initiation of therapy, the patient showed elevated levels of hsTnT (46 pg/ml) and increased NT-proBNP (723 ng/l). Clinically, she suffered from dyspnea (NYHA II-III) but denied typical chest pain.

**Table 1:** ICI-patient characteristics.

**Demog**	**Medical History**			**ICI regimen; number of doses received**	**Time to onset myocarditis; concurrent irAE**	**Myocarditis presentation**	**Immuno-modulators and other support (treatment sequence)**	**Outcome**
	**Cancer**	**Cardiovascular**	**Auto-immune**					
62y, M, 96 kg	Malignant melanoma, St.p. excision, Adjuvant chemotherapy with Pembrolizumab	- atrial fibrillation - St.p. mechanical aortic valve replacement	- bronchial asthma	Pembrolizumab (200 mg/3 weeks); two doses	27 days; myositis	Asymptomatic clinical course, hsTnT- and NTproBNP-elevation, coughing	250 mg prednisolone for 3 days with subsequent tapering	- No oncological sign of relapse - Persistent increased hsTnT and NT-proBNP levels - Normalization of initially significantly increased creatine kinase
70y, M, 120 kg	Hepatocellular carcinoma, Monotherapy Durvalumab	- CHD, PCI of RCA - Pulmary artery embolism - atrial fibrillation	none	Durvalumab (1,500 mg/ 4 weeks), two doses	39 days; myasthenia-like syndrome	Dyspnea (NYHA III- IV), fatique	500 mg prednisolone for 3 days with subsequent tapering, pyridostigmine	- Longterm mechanical ventilation, tracheostomy - rehabilitation after weaning - at least 4 months survival - Oncological reevaluation pending
74y, F, 62 kg	Adenocarcinoma of the uterus, metastasized peritoneal, vaginal, LN; St.p. hysterectomy prior to chemotherapy with Carboplatin and Paclitaxel 2018, Letrozol and Palbociclib 2018-01/2019, Pembrolizumab since 01/2019	- CHD without major lesions	none	Pembrolizumab (200 mg/ 3 weeks), 11 doses	271 days	Dyspnea (NYHA II), hsTnT- and NT-proBNP-elevation	100 mg prednisolone for 3 days with subsequent tapering	- Partial response peritoneal and LN - Declining hsTnT, stable dyspnea (NYHA II) - Alive at 3 months after the initial suspect of myocarditis

*Patient overview table, CHD, coronary heart disease; F, female; kg, kilogram; M, male; NT-proBNP, N-terminal pro–B-type natriuretic peptide; NYHA, New York Heart Association classification; hsTnT, high sensitive Troponin T; PCI, percutaneous coronary intervention; RCA, right coronary artery; St.p., status post; y, years*.

**Figure 1:**
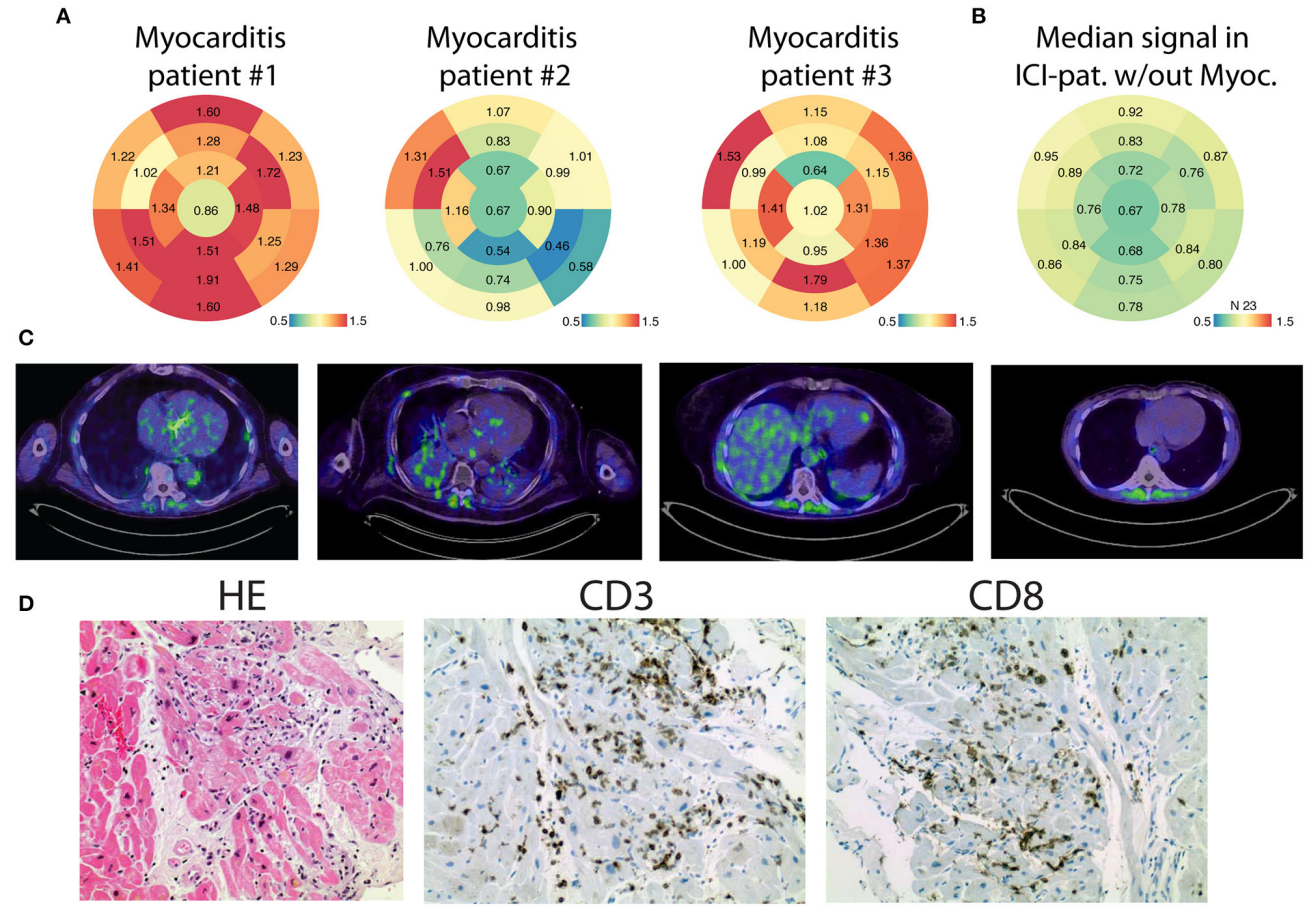
FAPI PET/CT illustrates ICI-associated myocarditis. **(A)** Bulls Eye Illustration of standardized uptake values (SUVs) showing their distribution in the myocardium of the left ventricle in 17 defined areas. The enrichment is shown for ICI-associated myocarditis patients #1–#3. **(B)** In comparison, the median signal of patients which have received immune checkpoint inhibitors (*n* = 23) without signs of myocarditis is summarized. **(C)** Exemplary images of ^68^Ga-FAPI PET/CT showing tracer uptake in the myocarditis patients' left ventricle and one example for the diagnostic findings in a non-myocarditis patient (right). **(D)** Exemplary histological sections of the left ventricle (HE: Hematoxylin staining, CD3- and CD8-immunostaining), confirming autoimmune myocarditis.

All patients fulfilled the criteria for definite ICI-associated myocarditis which have recently been suggested (4). Myocardial biopsy revealed CD3/CD8+ cells for patient #1 and patient #2. Exemplary images are shown in Figure 1D. In Patient #3, myocardial biopsy revealed <14 CD3/CD8+ cells/mm^2^. However, wall motion abnormalities in the echocardiography, elevated cardiac biomarker (hsTnT, NT-proBNP) and ST depressions in the ECG were found. In addition, the patient developed an autoimmune syndrome, including a general myositis with elevation of creatine kinase.

**Figure 2:**
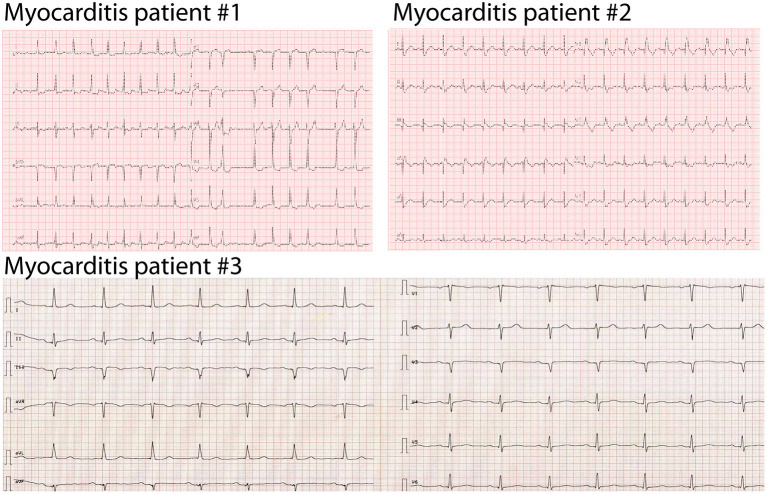
Initial presentation of ICI-associated myocarditis patients. Initial ECG at presentation in the hospital when ICI-associated myocarditis was diagnosed. Patient #1: 25 mm/s, patient #2: 25 mm/s, patient #2, patient #3: 50 mm/s.

All patients were treated with steroids, followed by tapering over several weeks. Holter ECG did not reveal higher grade arrhythmias, but the initial ECG showed T-wave inversions or ST depressions in all three patients (Figure 2). Echocardiography showed a preserved to slightly reduced LVEF in all patients. Cardiac MRI confirmed the preserved systolic ejection fraction (Supplementary Videos 1, 2) and an angiography was able to exclude significant ischemia in the area of FAPI enrichment (Supplementary Videos 3–5). Patient #2 received a coronary intervention of the right coronary artery. We did not observe late gadolineum enhancement nor tracer accumulation in the FAPI PET/CT in the inferior segments, but CD3/CD8+ cells in the myocardial biopsy. Patient #1 showed globally elevated T1-mapping, in patient #2 we found late gadolineum enhancement at the basal segments. The two patients did not show any signs of cardiac edema as evaluated by T2-mapping. In patient #3 no MRI was performed.

### Cardiac Imaging With the Use of the ^68^Ga-FAPI Tracer

Given that the results of the biopsies were not available immediately, we decided to perform a PET/CT with ^68^Gallium FAPI which enables the identification of activated fibroblasts. In addition to an uptake in the neoplastic tissues, the examination revealed an accumulation of the tracer either diffusely distributed in the left ventricle (patient #1), rather localized at the septal area (patient #2) or in the apical posterior wall of the left ventricle (patient #3) (Figures 1A,C).

We did not find comparable cardiac enrichments of the tracer in the control patients who received ICIs but who were not suspected of ICI-associated myocarditis. Even though some patients in our control group (patients #5, #6, #8, and #23) have shown slightly elevated SUVs in the heart, they have not shown obvious signs of either acute coronary syndrome, heart failure or myocarditis (*n* = 23, Figure 1B, Supplementary Figure 1). Regarding the clinical data of the patients, we found two patients with diabetes, five patients with atrial fibrillation and three patients with coronary heart disease in our non-myocarditis group. Three patients' history included chest radiation (Supplementary Table 1). Two patients in the control group with slightly elevated SUVs in the heart were diagnosed with coronary heart disease and one patient was subjected to chest radiation.

The median SUV in the myocarditis patients was 1.79 (IQR: 1.65, 1.85), whereas the median SUV in non-myocarditis patients was found to be 1.15 (IQR: 0.955, 1.52).

Thus, FAPI PET/CT allowed the identification of locally defined myocardial remodeling due to ICI-associated cardiac inflammation. Upon steroid treatment, cardiac troponin levels normalized in all three cases and the symptoms of dyspnea disappeared.

## Discussion

### The Challenge of the Early Diagnosis of ICI-Associated Myocarditis

Current cardiac imaging techniques fail to detect early stages of ICI-associated myocarditis, especially in the absence of functional impairments. Myocardial biopsies, currently considered as gold standard, do not allow an immediate conclusion. Biopsies further appear to be false-negative in some cases based on local differences in leukocyte infiltrations as seen in biopsies of patient #3 (3). This notion is supported by the diffuse pattern of ^68^Ga-FAPI enrichment, which was seen in this patient. Considering the increasing demand of ICIs and bearing the potentially life-threatening consequences of their use in mind, novel strategies for the diagnosis of ICI-associated myocarditis are needed.

### The Use of ^68^Ga-FAPI PET/CT in Cancer Staging and Cardiac Applications

Fibroblast activating protein (FAP) is known to be significantly upregulated in tissue remodeling, indicating tumor activity or fibrosis following the activation of fibroblasts (11, 12, 15). In terms of oncological staging, ^68^Ga-FAPI PET/CT showed a definite enrichment of the tracer in highly prevalent cancers and was beneficial for tumor characterization and discovering metastasis (16). It was able to show characterizations superior to FDG-PET analysis (13). A further application of ^68^Ga-FAPI PET/CT could be radioligand therapies of the neoplastic tissue as recently supposed (15).

Cardiac fibroblasts, as well as tumor fibroblasts, reliably express FAP during remodeling due to injury or disease [e.g., in dilated and hypertrophic cardiomyopathy or after myocardial infarction (14)]. Preclinical data indicates that selectively targeting FAP may serve as a therapeutical approach to inhibit cardiac fibrosis and to restore heart function after administration of Angiotensin II and Phenylephrine (9).

Here, we show the first use of ^68^Ga-FAPI PET/CT in the detection of myocardial alterations caused by ICI-associated myocarditis. Apart from myocardial infarction and coronary heart disease, there was no study yet to investigate its use in cardiac diseases. Derived from the pathomechanism, myocarditis in general and fibrosis in terms of cardiomyopathy might be detectable by FAPI PET/CT, as they are based on inflammation and tissue remodeling. Recently, it was shown that diabetes is associated with elevated tracer enrichment in FAPI PET/CT in the heart (17). This was associated with an enrichment of tracer accumulation within all segments of the left ventricle, whereas patients with ICI-associated myocarditis revealed a localized enrichment. Prior diagnosed coronary heart disease and chest radiation might be the reason why we observed tracer enrichment in some patients of our non-ICI-myocarditis group (patients #5, #6, #8, and #23).

The distribution of fibrosis, measured via late gadolineum enhancement in cardiac MRI, was shown to be diffusely distributed in the left ventricle in hypertrophic cardiomyopathy and focal in ischemic cardiomyopathy (18). Myocarditis of any cause, either autoimmune or virus-associated, is described to be a localized disease (19). In cardiac MRI, elevated T2-mapping or late gadolineum enhancement was found in <50% of patients with ICI-associated myocarditis (20). This may be the reason why we were not able to correlate the MRI results with enrichment in FAPI PET/CT. Summarizing the limited clinical data that is published on ICI-associated myocarditis, the disease is supposed to show locally defined lymphocyte infiltrations as well (21).

Thus, we can link the locally defined tracer enrichment seen in the present FAPI images to the current knowledge of ICI-associated myocarditis. Further studies need to evaluate if FAPI-guided biopsies can reveal higher rates of positive CD3/CD8 immunostaining.

Since the onset of ICI-associated myocarditis is hard to define and the disease has a supposed transient character, mild intensities of the enrichment in the present study may be explained by the timing of the scan. However, in every patient with evidence of myocarditis, we see median SUVs above the median of our control group and in 2/3 patients immunostaining was able to detect relevant lymphocyte infiltration. The strength of this approach is a potential interdisciplinary evaluation of cancer staging and detection of myocarditis as an adverse effect at the same time. As recently shown in glioblastoma, FAPI enrichments are tissue-specific and do not have a strong correlation with blood flow and perfusion (22).

## Conclusion

Novel approaches for sensitive, non-invasive diagnostics are needed because myocardial biopsies require cardiac catheterization and results are not immediately available. In addition, in many cases they appear to be false-negative. We propose that ^68^Ga-FAPI PET/CT could be a non-invasive, unbiased method for the diagnosis of ICI-associated myocarditis.

Further studies need to address the predictive value and best time-window to diagnose ICI-associated myocarditis via FAPI PET/CT.

## Limitations

Some limitations of this work need to be acknowledged. Due to the rare manifestation of ICI-associated myocarditis, we could only enroll a relatively small number of patients with evidence of ICI-associated myocarditis.

In our control group, containing patients receiving ICIs without any evidence of myocarditis or further cardiac pathologies, we were not able to perform a detailed cardiac assessment containing cardiac catherization and cardiac MRI as shown for the three cases of ICI-associated myocarditis. Anamnestically, there were no hints for the occurrence of a cardiac disease in those patients. FAPI PET/CT was done at one time point. Thus, we bear in mind the possibility of changes in the tracer enrichment during the time course of ICI therapy.

## Data Availability Statement

The original contributions presented in the study are included in the article/Supplementary Materials, further inquiries can be directed to the corresponding author/s.

## Ethics Statement

The studies involving human participants were reviewed and approved by the ethics committee of the Medical Faculty of University Heidelberg. The patients/participants provided their written informed consent to participate in this study.

## Author Contributions

DF and LHL conceived and designed the study and wrote the manuscript. DF, MH, EH, HK, UH, FL, and LHL analyzed and interpreted the data. DF, LL, MH, UH, FL, and LHL drafted the manuscript and revised it critically. All authors have read and approved the manuscript.

## Conflict of Interest

LHL has served on the advisory board for Daiichi Sankyio, Senaca and Servier, and received speakers' honoraria from Novartis and MSD. DF, MH, UH, HK and LHL have filed a patent for the use of FAPI imaging for the detection of pathological cardiac remodeling. The remaining authors declare that the research was conducted in the absence of any commercial or financial relationships that could be construed as a potential conflict of interest.
