# Supplementary material for: Early Detection of Checkpoint Inhibitor-Associated Myocarditis Using 68Ga-FAPI PET/CT
Source: Front Cardiovasc Med. 2021 Feb 25;8:614997. doi: 10.3389/fcvm.2021.614997 (PMC7946849; doi:10.3389/fcvm.2021.614997)
Supplement: Supplementary file 1 [file Data_Sheet_1.docx]

**Early detection of checkpoint inhibitor-induced myocarditis using ^68^Ga-FAPI PET/CT**

Daniel Finke ^1,2^, Markus B. Heckmann, MD ^1,2^, Esther Herpel, MD ^3^, Hugo A. Katus, MD, PhD ^1,2^, Uwe Haberkorn, MD ^4,5,6^, Florian Leuschner, MD ^1,2^, Lorenz H. Lehmann, MD ^1,2*^

^1^ Department of Cardiology, Uni­versity Hospital Heidelberg, 69120 Heidelberg, Germany

^2^ DZHK (German Centre for Cardiovascular Research), partner site Heidelberg/Mannheim, Germany

^3^Department of Pathology, University Hospital Heidelberg, 69120 Heidelberg, Germany

^4^ Department of Nuclear Medicine, University Hospital Heidelberg, 69120 Heidelberg, Germany

^5^ Clinical Cooperation Unit Nuclear Medicine, DKFZ, 69120 Heidelberg, Germany

^6^ Translational Lung Research Center Heidelberg (TLRC), German Center for Lung Research (DZL), Heidelberg, Germany

**Supplemental Figure 1:** FAPI PET/CT tracer enrichment in patients treated with ICIs without signs of myocarditis

**
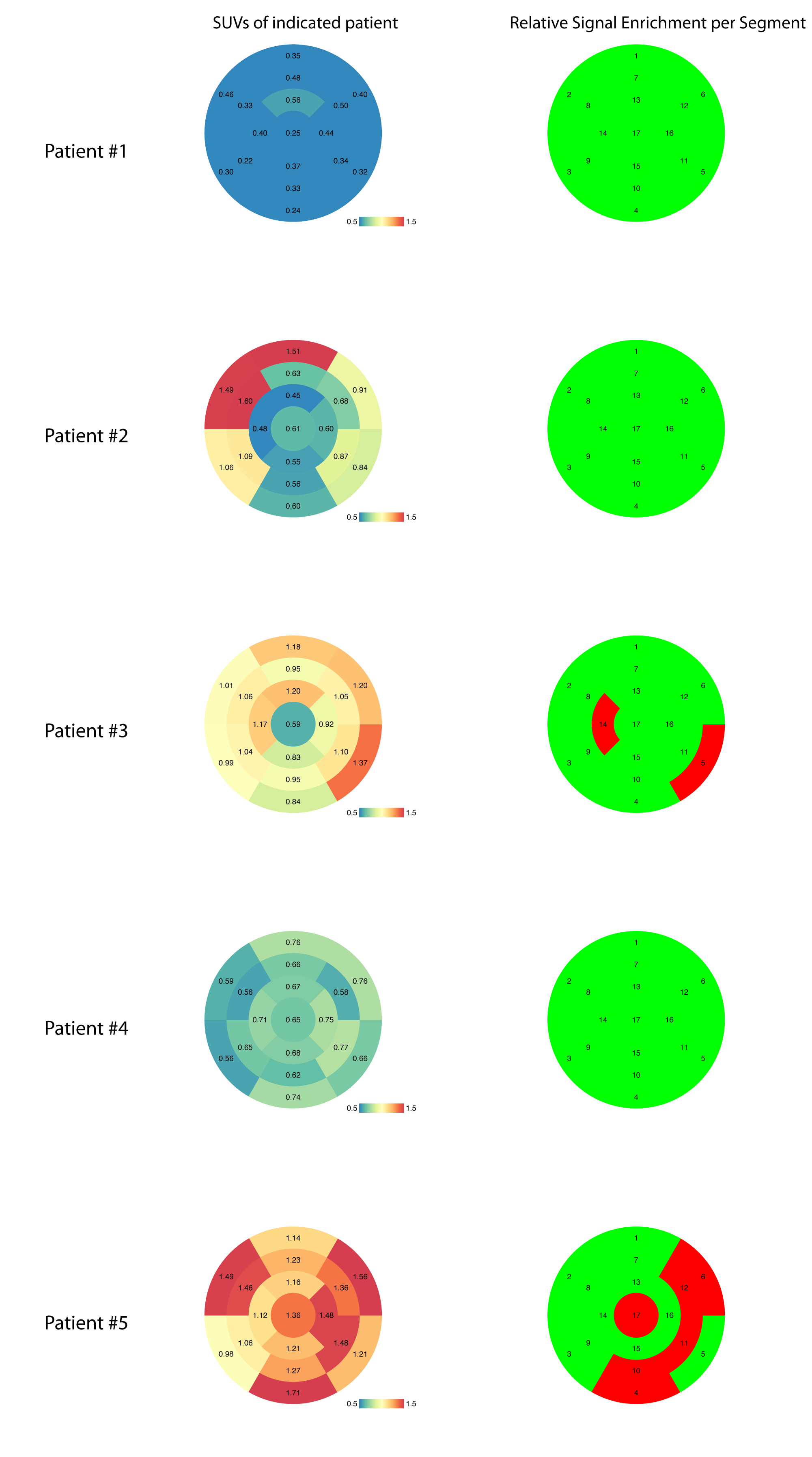
**

**
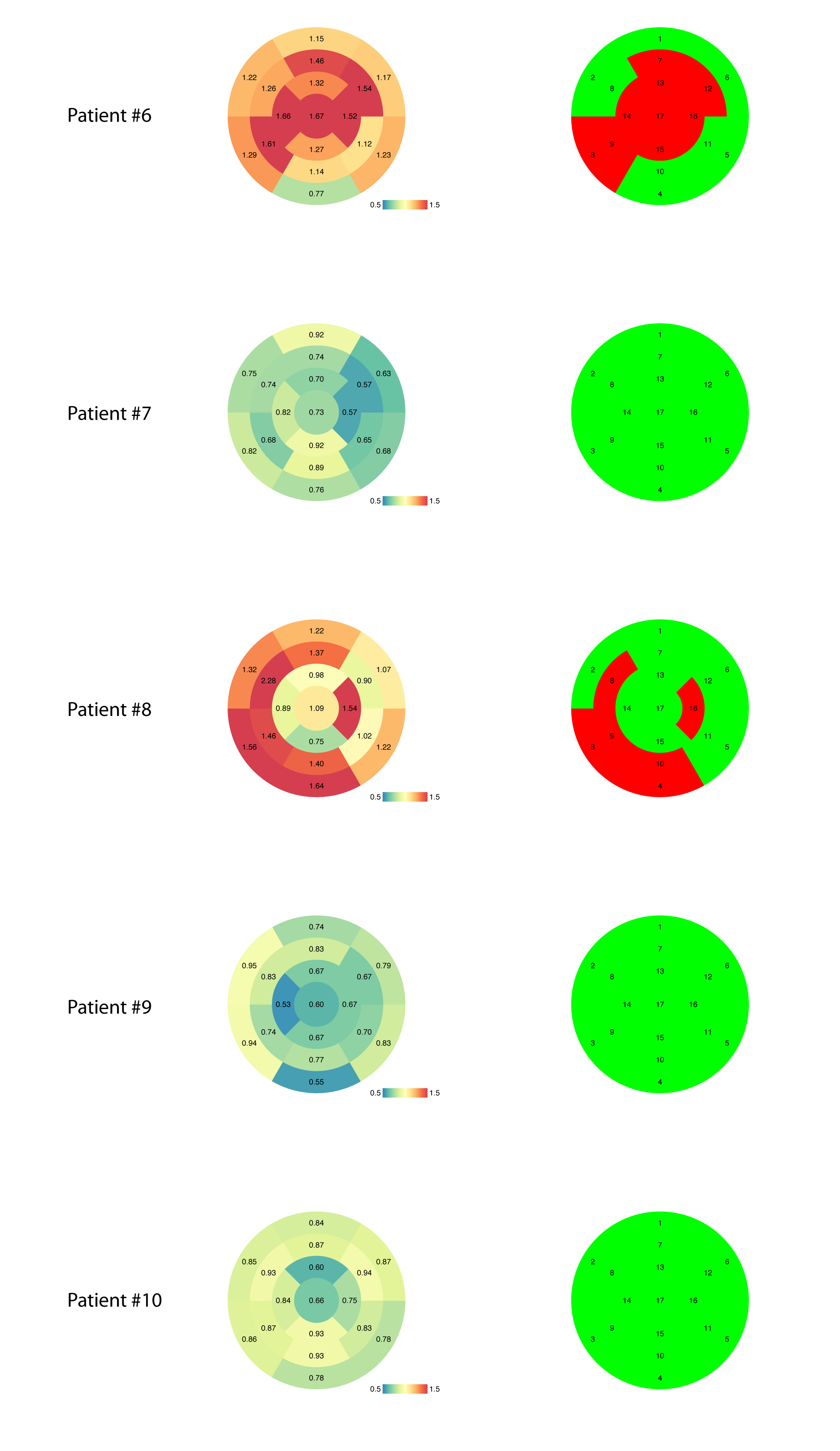
**

**
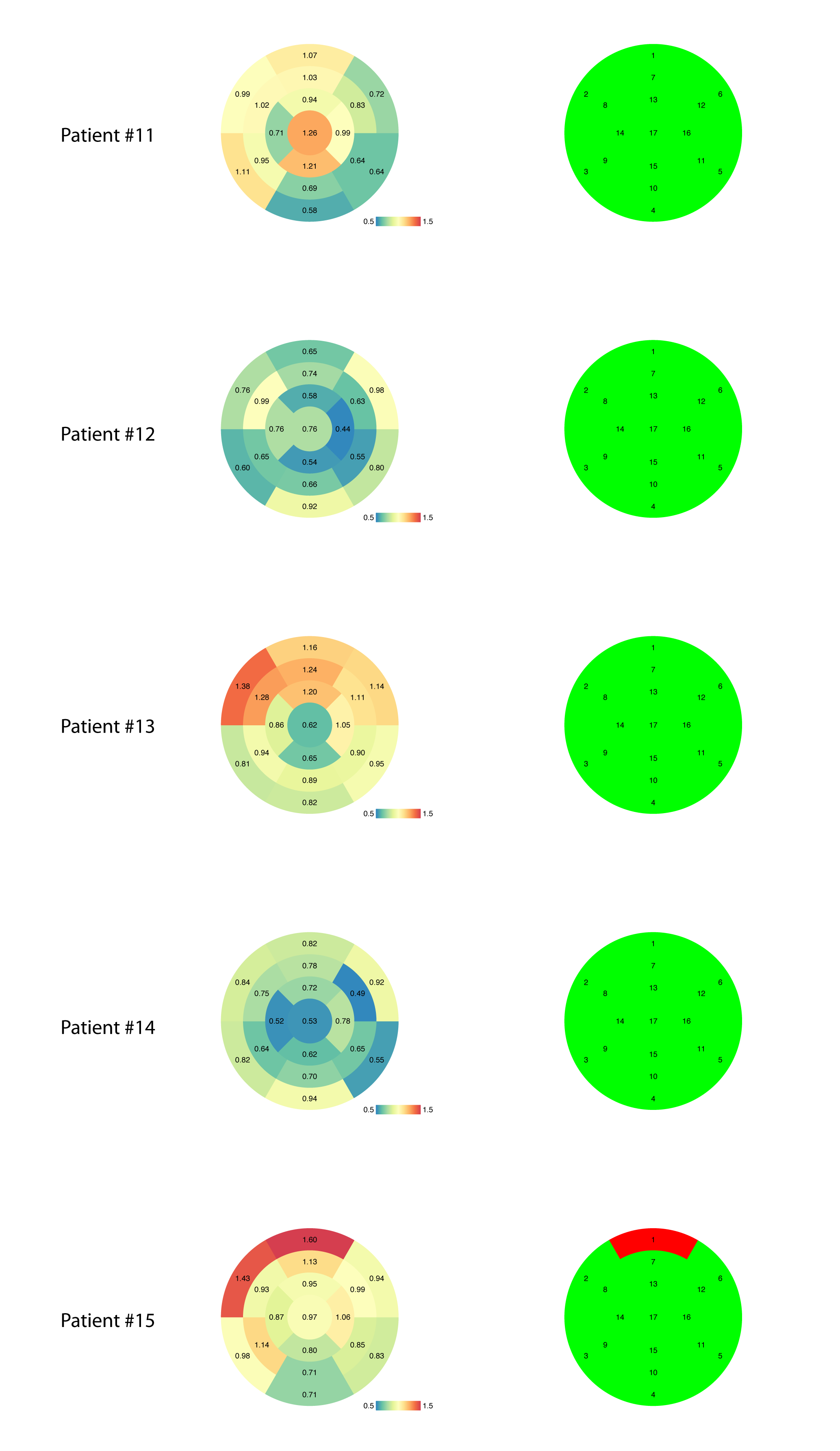
**

**
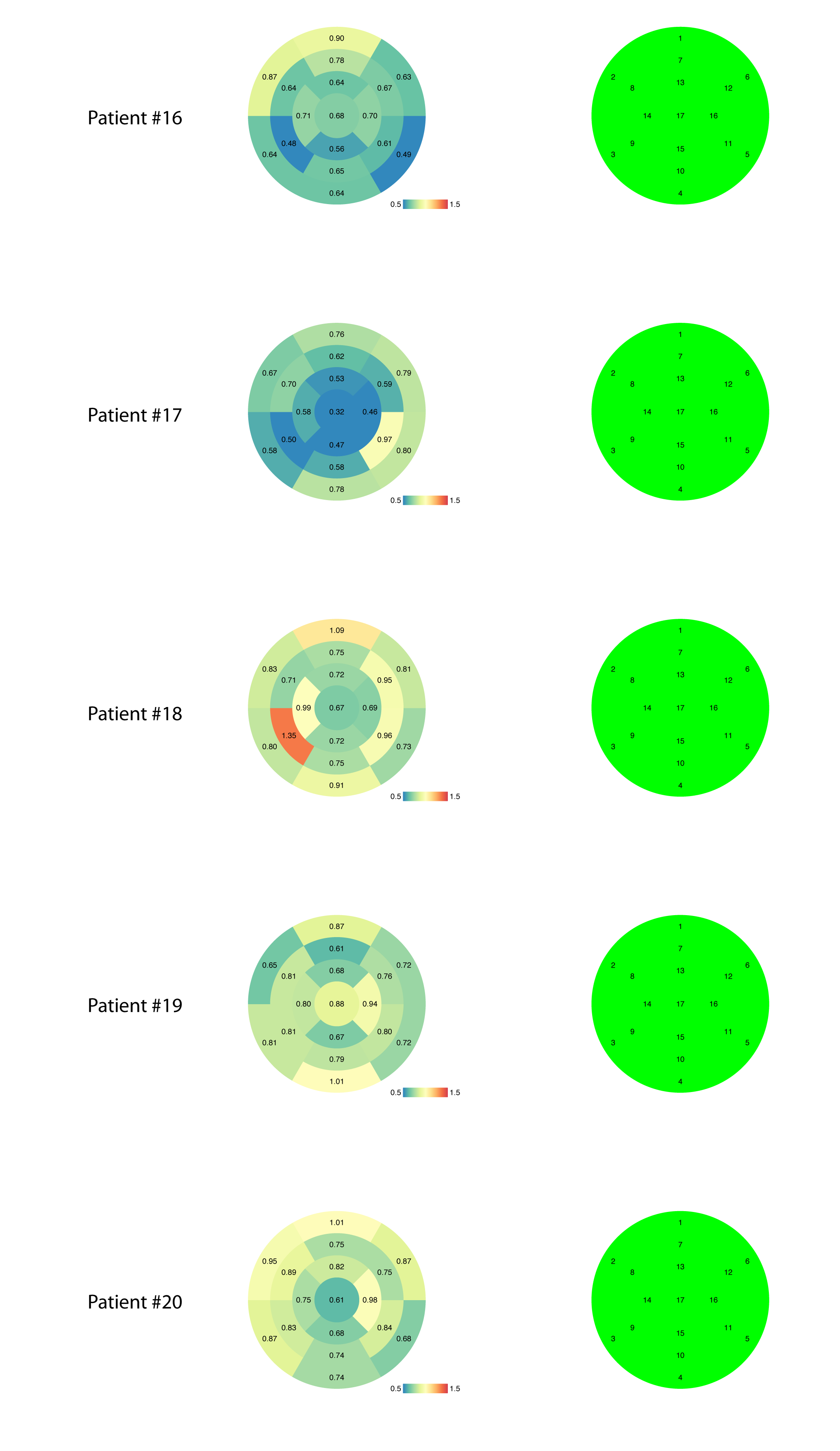
**

**
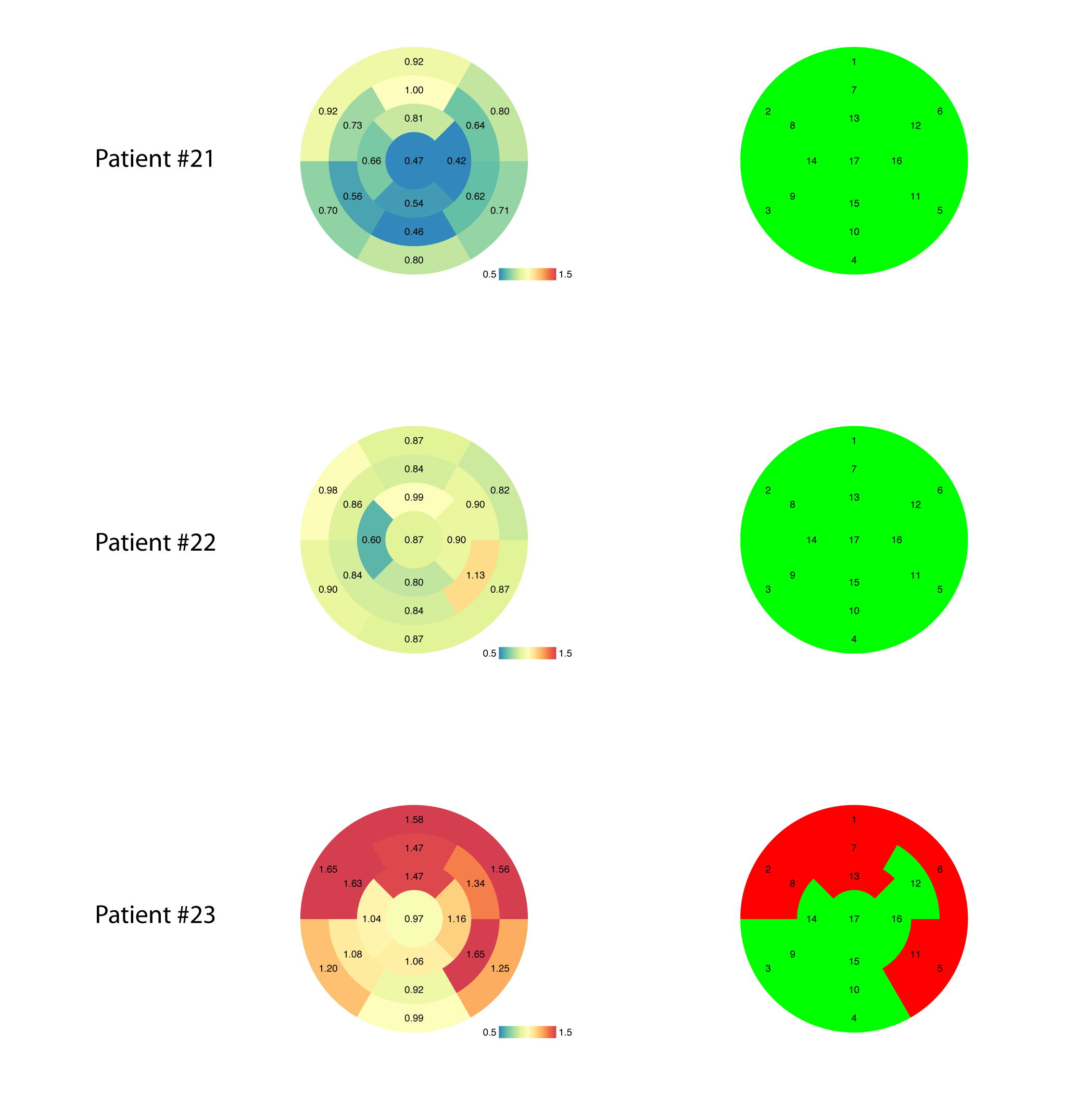
**

**Supplemental Figure 1: FAPI PET/CT tracer enrichment in patients treated with ICIs without signs of myocarditis**

Bulls Eye Illustration of standardized uptake values (SUVs) showing their distribution in the myocardium of the left ventricle in 17 defined areas. The enrichment is shown for ICI-treated patients #1-#23 without any signs of myocarditis. Color code as indicated. In a second Bulls Eye Illustration (right) FAPI signals above the 95^th^ percentile of the cohort are marked in red. Signals below are colored in green.

|  | **Total (n=26)** | **Myocarditis patients (n=3)** | **Non-myocarditis patients (n=23)** |
| --- | --- | --- | --- |
| Age [years] | 67.4 [49.6; 74.5] | 70 [66, 72] | 66.9 [47.5; 75.9] |
| Male gender | 15 (57.7%) | 2 (66.7%) | 13 (56.2%) |
| **Cardiovascular risk factors** |  |  |  |
| Arterial hypertension | 9 (34.6%) | 3 (100%) | 6 (26.1%) |
| Diabetes | 3 (11.5%) | 1 (33.3%) | 2 (8.6%) |
| BMI | 21.7 [19.8; 25.4] | 25.5 [24.4; 31.3] | 21.5 [19.4; 24.0] |
| Smoking | 1 (3.8%) | 0 (0%) | 1 (3.8%) |
|  |  |  |  |
| **Cardiovascular disease** |  |  |  |
| Coronary Heart Disease | 4 (15.4%) | 1 (33.3%) | 3 (13.0%) |
| Atrial fibrillation | 7 (26.9%) | 2 (66.7%) | 5 (21.7%) |
|  |  |  |  |
| **Therapy** |  |  |  |
| Additional chemotherapy | 23 (88.4%) | 1 (33.3%) | 22 (95.6%) |
| Chest Radiation | 3 (11.5%) | 0 (0%) | 3 (13.0%) |
| ACE-inhibitor | 7 (26.9%) | 3 (100%) | 4 (17.4%) |
| Beta blocker | 4 (15.4%) | 3 (100%) | 1 (4.35) |
| ASA | 3 (11.5%) | 2 (66.7%) | 1 (4.3%) |
| Statins | 4 (15.4%) | 2 (66.7%) | 2 (8.7%) |

**Supplemental Table 1: Patient characteristics of patients with and without ICI-associated myocarditis**

ACE: Angiotensin converting enzyme, ASA:acetylsalicylic acid, BMI: Body Mass Index
